# How to Prevent After-Pains*Vol. VI, p. 60, International Cyclopedia of Surg.

**Published:** 1891-12

**Authors:** T. J. Bennett

**Affiliations:** Austin


### HOW TO PREVENT AFTER-PAINS.*

T. J. BENNETT, M. D., AUSTIN.

Read at 22nd Annual Meeting State Medical Association.

IT IS well known that the after-pains are produced by the contractions of the uterus, in the effort to expel coagula, formed from blood poured out from the site of the detached placenta; it

*Vol. VI, p. 60, International Cyclopedia of Surg.

is also well known and advised, assuming these premises, that the expulsion of coagula and prevention of others can be accomplished by a uniform and permanent contraction of this organ. It arrests the hemorrhage and therefore prevents the formation of clots.

It would seem therefore that the desideratum to be attained would be some method of procedure whereby this uniform and permanent contraction can be brought about.

The object of this brief paper is to give the results of the writer's observation and experience in twelve or fifteen well selected cases, during the past two years.

After the third stage of labor is over, introduce two or three fingers into the vagina, and gently manipulate the vaginal portion of the uterus in a way to secure, as nearly as possible, the closure of the gaping cervix and os; employing the other hand over the hypogastrium, merely to steady the organ. The manipulation necessary to effect this object is not painful; it consists in merely sweeping the fingers around the vaginal portion two or three times, at the same time alternately pressing the parts hard enough to touch the portion already contracted; this at once stimulates a further contraction of the organ, and the firmness in the uterine wall will be noticed to take place at, and originate from, the already hardened portion and extend outward toward the rim.

It is a common observation to find the womb, after the third stage of labor, globular in shape. This is due to the fundus alone being contracted. The whole vaginal portion, and especially the immediate os is, at this time, relaxed and flabby almost beyond recognition by touch, but a little effort at drawing this loose structure together will show that firmness in the tissues is gradually taking place. The circular fibres of the os do not contract to the same extent that those higher up do, owing to the fact that the temporary paralysis from long pressure and distention is greater at the mouth than elsewhere.

After a brief manipulation as described, it will be found that the uterus, instead of being globular in shape, as before, has assumed an oblong, or pear-shape; in other words, a cervix has been formed, and the process of involution has been advanced some two or three days. The sensation to the touch in examination after this operation is about like that imparted if examined in two or three days after delivery ordinarily.

*Published in Transactions Texas State Medical Association, 1891.

Hemorrhage, unless there be laceration, is always from the site of the detached placenta; and in a majority of cases, this point is the fundus. If firm contraction of this portion can be secured there will be no hemorrhage, therefore no clots and, ergo, no after-pains.

It is evident that additional force is secured to the fundus by causing the vaginal portion to similarly contract, and this I think explains the success of the procedure. The advantages gained in having secured this uniform contraction, are numerous and must be apparent. The danger of post-partum hemorrhage is thereby greatly diminished; the lochial discharge is abbreviated several days, and the patient, relieved from the harrowing and wearing after-pains, which, by some are more dreaded than those of actual labor, gets the rest that is then most needed. These are the advantages secured to the patient; while the accoucheur, feeling that all is well, is relieved of the unpleasant apprehensions of a sudden recall to find perhaps a dangerous or alarming flooding.

These may be said to be small things to write about, but it is attention to just such features in practice that often gives good results and no small amount of satisfaction to both physician and client.

